# What is the carbon footprint of primary care practices? A retrospective life-cycle analysis in Switzerland

**DOI:** 10.1186/s12940-021-00814-y

**Published:** 2022-01-04

**Authors:** John Nicolet, Yolanda Mueller, Paola Paruta, Julien Boucher, Nicolas Senn

**Affiliations:** 1 Department of Family Medicine, Center for Primary Care and Public Health (Unisanté), University of Lausanne, Lausanne, Switzerland; 2 EA – Environmental Action, Lausanne, Switzerland; 3 School of Management and Engineering Vaud, HES-SO Yverdon-les-Bains, Switzerland

**Keywords:** Primary care, Practices, Public health, Carbon footprint, Climate change

## Abstract

**Background:**

The medical field causes significant environmental impact. Reduction of the primary care practice carbon footprint could contribute to decreasing global carbon emissions. This study aims to quantify the average carbon footprint of a primary care consultation, describe differences between primary care practices (best, worst and average performing) in western Switzerland and identify opportunities for mitigation.

**Methods:**

We conducted a retrospective carbon footprint analysis of ten private practices over the year 2018. We used life-cycle analysis to estimate carbon emissions of each sector, from manufacture to disposal, expressing results as CO_2_ equivalents per average consultation and practice. We then modelled an average and theoretical best- case and worst-case practices. Collected data included invoices, medical and furniture inventories, heating and power supply, staff and patient transport, laboratory analyses (in/out-house) waste quantities and management costs.

**Results:**

An average medical consultation generated 4.8 kg of CO_2_eq and overall, an average practice produced 30 tons of CO_2_eq per year, with 45.7% for staff and patient transport and 29.8% for heating. Medical consumables produced 5.5% of CO_2_eq emissions, while in-house laboratory and X-rays contributed less than 1% each. Emergency analyses requiring courier transport caused 5.8% of all emissions. Support activities generated 82.6% of the total CO_2_eq. Simulation of best- and worst-case scenarios resulted in a ten-fold variation in CO_2_eq emissions.

**Conclusion:**

Optimizing structural and organisational aspects of practice work could have a major impact on the carbon footprint of primary care practices without large-scale changes in medical activities.

## Introduction & background

The Intergovernmental Panel on Climate Change (IPCC), warned that global warming will significantly affect hundreds of millions of people [1], but also found that mitigation options are available in every major sector and on every scale, from local to international. The medical scientific community has increasingly urged for emergency action on climate change [2, 3]. In this context, *The Lancet Countdown on health and climate change* started to review annually forty-one indicators that explore the relation between health and climate change and to assess the progress made by governments towards their engagement to the 2015 Paris agreement [4–6].

Among the forty-one indicators, one focused on the mitigation of health care sector emissions. Hospitals, medical devices and procedures or national health care systems as a whole have had their carbon footprints examined. Estimations exist for all OECD countries, while more-detailed studies are available for Australia, China, Japan and the United Kingdom [7–12]. As an example, 10 % of the US-American greenhouse-gas (GHG) emission is generated by its health care sector (directly and indirectly), which represents almost two tons of CO_2_eq per inhabitant annually [13]. In Switzerland, this same sector is estimated to produce one ton per inhabitant annually [6]. The concept of carbon budget helps to put these numbers into perspective. The carbon budget is the estimated quantity of GHG emissions that humanity should not exceed to keep the global temperature below a certain limits, 1.5 °C in the Paris Agreement. In Switzerland, this budget is evaluated at 0.6 tons of CO_2_eq per inhabitant, accounting for previous emissions and further population growth to have a 50% chance of staying below a 2 °C increase [14].. Based on these estimates, the Swiss healthcare system surpasses the national carbon budget by almost two times.

While the first steps of measurement of greenhouse gas emissions in healthcare has progressed, initiatives to reduce them appear on several levels [15–21]. Despite the fact that primary care is a significant part of the health care system, little has been done to address its specific footprint. Although, the National Health Service in England has been studying its carbon footprint for more than a decade [16]. However, none of the studies measured the emissions of primary care practice by assessing them in detail directly on site rather than estimating from environmentally extended economic input-output life cycle assessment models.

In addition, primary care systems differ from country to country, for example in terms of practice equipment (laboratory or X-ray) or task distribution between primary and secondary care. Thus, mitigation of the activities of primary care practice needs to be addressed specifically and on a national level.

In Switzerland, more than 8000 primary care providers work in about 3000 practices, mainly organized as small and independent businesses. They provide more than 13.8 million medical consultations per year [22]. On a daily basis, primary care providers use medical equipment and consumables not found in other businesses: many use in-house X-ray equipment (57%) and laboratory tests (66%) [22].

This study aims to quantify the average carbon footprint of a primary care consultation, describe differences between primary care practices (best, worst and average performing) in western Switzerland and identify opportunities for mitigation.

The assessment of GHG emissions in a decentralized health system such as Switzerland, where primary care is delivered mainly in private practices may be of interest for other countries having similar organisation. Furthermore, a bottom-up approach with a systematic description of the sources of emission is flexible and detailed allowing projecting to other primary care settings.

## Methods

The study consisted of a carbon footprint analysis of ten private primary care practices in western Switzerland. The methodology complied to the Life Cycle Assessment (LCA) approach [23] but focussed solely on the GWP100 metric for the assessment of climate change impacts [24]. All sources of GHG emissions were included and converted to their CO_2_ equivalent, mentioned as CO_2_eq throughout this article. The approach considers all life stages of a product or activity, from its manufacture, usage, and maintenance to disposal.

For the purpose of this study, we use the three Tiers to report the results of the LCA: 1. Practice activities, 2. Patient transport and 3. External stakeholder activities. We further subdivided Tiers into twelve domains, as detailed in Table 1. This tiered approach intends to allocate the impacts to the key stakeholders involved, based on their role and responsibilities. This subdivision is further used to interpret the results and suggest possible actions.

**Table 1:** Tiers and domains: description, sources and examples

LCA Tiers	Domain	Number of items listed	Examples of items	Type	Data sources
1	Medical equipment	39	Stethoscope, thermometer, sphygmomanometer, scales, examination bed, needle, syringe, flashlight, saturometer, otoscope, ECG and x-ray devices	Medical	Practice inventory
1	Non-medical equipment	20	Computer, printer, desk, chair, table	Support	Practice inventory
1	Medical consumables	57	Bandage, compress, disinfectant, gloves, mask, tongue depressor, scalpel, swab test	Medical	Invoicing and billing
1	Non-medical consumables	15	Ink, toner, battery, paper, paper towels, plastic cups	Support	Invoicing and billing
1	Waste		General (non-recyclable) waste, paper, plastic, glass, hazardous waste	Support	Invoicing and billing, staff observation
1	Staff mobility		Number of staff, mode of transportation, time and distance to the practice or to training place	Support	Survey
2	Patient mobility		Mode of transportation, time and distance to the practice	Support	Survey
3	Courier mobility		Means of transport, journey type, location of dispatch centre	Medical	Invoicing and billing
1	Internal laboratory analyses		Equipment characteristics, usage patterns, number and types of analyses	Medical	Practice inventory, invoicing and billing
3	External laboratory analyses		Number and types of analyses, details of external laboratories commissioned	Medical	Invoicing and billing
1	Infrastructure		Surface area, date of founding, heating-system; type and consumption, running water consumption	Support	Invoicing and billing
1	Electricity		Electricity consumption and source (renewable or not), included energy consumption of in-house computer server, and x-ray device if present	Support	Invoicing and billing

*Note*: *LCA :* Lifecycle analysis

Tier 1: Practice activities GHG emission

Tier 2: Patient transport GHG emission

Tier 3: External stakeholder activities

For each domain, we defined a generic version of its items and activities that then applied this to all practices. For example, we estimated that stethoscopes, examining tables or laptops from different practices did not differ much from one another in terms of their carbon footprint and considered them respectively equivalent. This allowed a rapid on-sight listing of the items of each practice. Likewise, we treated medical activities that require consumables, such as blood sampling, urine testing, swab performing or electrocardiogram recording as conducted in a similar manner, using the same consumables in each practice.

In spring 2019, an invitation was sent to the teaching body of primary care physicians (50 people) of the University of Lausanne (western Switzerland) by e-mail. Volunteers had to agree to have their medical practice examined, in terms of the building, furniture, mobility of staff members and patients, and invoicing and billing. In this exploratory analysis, we limited the number of practices to ten. We aimed for a reasoned sample of practices in western Switzerland according to region, urban or rural setting, practice size, and age and gender of the physician. We excluded practices having many more physicians (> 10), than the Swiss average of 4.2 physicians, or practices not having mainly primary care specialists [22, 25].

Investigators performed an on-site data collection of practice and staff characteristics. Personal staff information collected included age, gender, professional level, transportation means, and time to and distance from the practice. We then meticulously referenced all practices equipment. In addition, we reviewed in detail all invoices and billing data to record practice activities, including energy consumption (electric, solar, gas, oil and water), sterilization, outsourcing processes, consumable use, amount of waste, the premises plan, and the number of medical consultations and samples (blood, urine, biopsy and swabs) tested in and out-house in the past year. We visited the most-frequently commissioned external laboratory to estimate sample analysis footprints. Data included lab machine life cycles, waste, information technology (IT) infrastructure, electrical consumption and courier transport. We did not collect prescription data in this study. Finally, we estimated the mode of transport patients used to get to the practice using a survey given to the patients either attending on the day of data collection only (for six practices), or to all those attending over a week (for four practices). The retrospective data collection timeframe was the year 2018, except for one practice, which was 2017 due to data unavailability for 2018.

We employed *Ecoinvent* [26] as the main life cycle inventory database for the data analysis, which allowed us to obtain the carbon footprint of each activity and item listed in the study. An Excel tool was developed for running the calculation of the process-based LCA. The durable equipment were also modelled based on process LCA, by estimating the average mass of the key components (electronic, metal and plastic) and a lifetime. The embodied emissions in the building were considered out of scope. The energy mix used is the average supply mix of Switzerland, except for the practices benefiting from a green mix or equipped with solar panels, where in both cases a more specific mix has been used.

For the analysis, first we added up the carbon footprints of each of the twelve domains (Table 1) to determine the annual carbon footprint of each practice. Then, we divided each practice footprint by its yearly number of consultations to obtain the average carbon footprint per consultation for each practice. After that, we combined the data of the ten practices to determine the characteristics of an average practice, and detailed its carbon footprint by domain and consultation.

Subsequently, we modelled a theoretical “lowest carbon emitting practice”’ by selecting the domains with the lowest carbon impact from each of the ten practices. By contrast, we also combined the highest footprints by domain to obtain a theoretical highest carbon emitting practice. Comparing the two models enabled us to suggest potential mitigation possibilities.

## Results

Ten medical practices were included in the study from sixteen applications received after the call. They were located in sub-urban (five practices), urban (four) or rural (one) or domains. Their surfaces ranged between 107 and 180 m^2^ except for two practices of 600 m^2^. The practices employed between 0.8 and 4.0 full-time equivalent non-physician staff and from 0.8–3.5 full-time equivalent physicians. All held a General & Internal medicine post-graduate specialisation title according to the Swiss medical association (https://www.siwf.ch/fr/formation-postgraduee/titres-specialiste-formations/medecine-interne-generale.cfm). Each practice provided between 1558 and 10,560 consultations over a year, corresponding to between 1558 and 3813 consultations per physician. From the 10 practices, combining 79 staff members (physicians + non-physicians), 53 (67.0%) answered the cross-sectional survey. Forty-one of them were women (77.3%). Twenty-seven (50.9%) worked as a practice assistant, twenty-two as a physician (41.5%), and the others (7.6%) as nurses, accountants or laboratory technicians.

Based on these characteristics, we defined an average practice as consisting of two full-time physicians and two full-time practice assistants, working in a 207-m^2^ premises. Together, it provides 6273 consultations per year, equivalent to 27 consultations per day (considering time-off and holidays of staff). See Table 2 for details.

**Table 2:** Practices characteristics

	Minimum	Maximum	Average practice
Premises surface	107 m^2^	600 m^2^	**207 m**^**2**^
Number of non-physician staff (full-time equivalent)	0.8 pers.	4 pers.	**2 pers.**
Number of physician staff (full-time equivalent)	0.8 pers.	3.5 pers.	**2 pers.**
Consultations provided annually by practice	1558	10′560	**6273**
Internal laboratory	8 out of 10 practices had one		**Yes**
Internal X-ray device	4 out of 10 practices had one		**Yes**
Ownership / rental	4 out of 10 own their practice		**Ownership**

Overall, the average practice produced 30.5 CO_2_eq tons for the year 2018, as detailed in Table 3, corresponding to an average of 4.8 CO_2_eq kg per consultation. More than half (55.5%) of the carbon footprint was due to mobility. This included patient mobility (33.2%) first-place for the highest carbon footprint, staff mobility (12.5%) in third-place and courier mobility (9.8%) in fourth-place. Second-place was the heating system, producing 9.1 CO_2_eq tons, which represented 29.8% of the whole footprint. Medical consumables came fifth with 1.7 CO_2_eq tons (5.5%), followed by non-medical equipment with 1.2 CO_2_eq tons (4.1%). The last six domains were waste disposal (1.6%), external laboratory analysis (1.2%), non-medical consumables (1.1%), in-house laboratory (0.5%) and medical equipment (0.4%). Electricity consumption came last place and accounted for 0.3% of the total footprint.

**Table 3:** Yearly carbon footprint of the average practice

Rank	Domain	Sub-domain	Carbon footprint CO_2_eq kg	Proportion by domain	Proportion of total footprint
**1**	**Patient mobility**		**10145**	100%	**33.2%**
**2**	**Heating system**		**9106**	100%	**29.8%**
**3**	**Staff mobility**		**3816**	100%	**12.5%**
**4**	**Courier mobility**		**2997**	100%	**9.8%**
		Regular couriers (blood samples)	638	21.2%	
		On-call couriers (blood samples)	1747	58.0%	
		On-call couriers (special waste)	613	20.4%	
**5**	**Medical consumables**		**1678**	100%	**5.5%**
		Bandages and compresses	1051	62.6%	
		Blood sampling materials: needle, tube, etc.	211	12.5%	
		Bed sheets (paper)	147	8.7%	
		Gloves	73	4.3%	
		Urinary rapid test	39	2.3%	
		Disinfectant	33	1.9%	
		Others, e.g.: mask, scalpel, swab test, shot material, tongue depressor, electrode, oxygen bottle	124	7.3%	
**6**	**Non-medical equipment**		**1239**	100%	**4.1%**
		Computer - 4 yr. of use	714	57.5%	
		Furniture: desk, chair, cupboard, etc. - 10 yr. of use	234	19%	
		Telephone - 3 yr. of use	212	18.8%	
		Printer - 5 yr. of use	74	5.9%	
		Other electronic devices	6	0.4%	
**7**	**Waste**		**491**	100%	**1.6%**
		General waste	321	65%	
		Special waste (radioactive)	164	33%	
		Paper waste	6	1%	
**8**	**External laboratory analysis**		**370**	100%	**1.2%**
**9**	**Non-medical consumables**		**338**	100%	**1.1 %**
		Paper	117	34.7%	
		Toner /ink	79	23.5%	
		Paper towels	77	22.9%	
		Postal service	64	18.9%	
**10**	**In-house laboratory**		**152**	100%	**0.5%**
**11**	**Medical equipment**		**110**	100%	**0.4%**
		Examination beds - 20 yr. of use	87	78.8%	
		Tensiometers - 5 yr. of use	16	14.2%	
		Electrocardiogram device, thermometer, tuning fork, glucometer, otoscope, scale, dermatoscope, flashlight, stethoscope, demonstration models	8	7.1%	
**12**	**Electricity**		**95**	100%	**0.3%**
	**TOTAL**		**30,538**		**100%**

*Note*: In-house x-ray emission are included in electricity and medical equipment

In this study, 63% of staff and 75% of patients travelled by car to the practice. However, this mode of transportation represented more than 99% of the staff and patient mobility footprint. For courier mobility, 78.7% of the footprint was related to the on-call couriers, who performed trips back and forth to dispatch centres. By contrast, regular couriers usually did round trips that served several practices.

The medical consumables domain contained the largest number of different items (57 considered) with bandages and compresses accounting for 62.6% of this domain’s footprint, followed by blood sampling material (12.6%), medical bed paper sheets (8.8%), gloves (4.3%), the rapid urinary test (2.3%) and disinfectant (2.0%). The rest of the items accounted for less than 1% each.

For non-medical equipment, electronic devices accounted for 81.1% of the footprint, with furniture amounting to 18.9%. The medical equipment footprint was mainly due to examination beds (78.8%). Tensiometers represented 14.2%. Small medical devices (e.g. ECG, thermometers, otoscopes, balances) and tools (e.g. stethoscopes, reflex hammers and tuning forks) composed the remaining 7.1%.

**Fig. 1:**
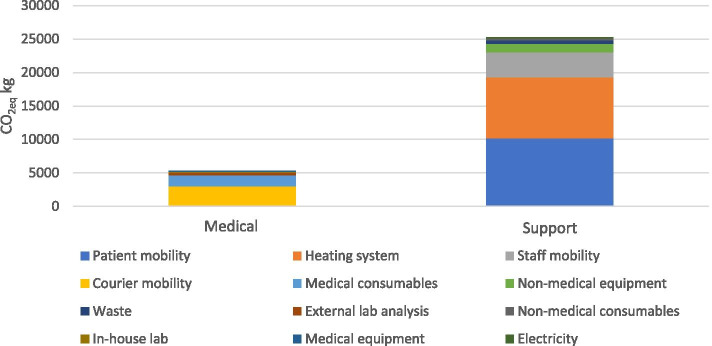
Yearly carbon footprint of the average practice by linkage with medical activities

Figure 1 classifies the footprint of the twelve domains for the medical and support activities of the average practice. We observed that 82.6% (25′231CO_2_eq kg) of the footprint was due to support activities.

**Fig. 2:**
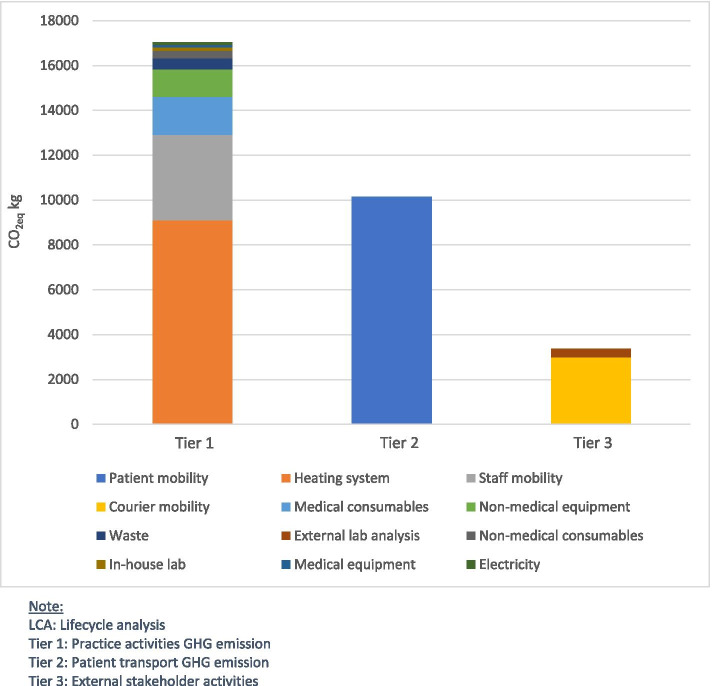
Yearly carbon footprint of the average practice by tiers

Activities directly conducted by the practice staff (Tier 1) totalled 55.8% (17′026.0 CO_2_eq kg) of the practice’s footprint as shown in Fig. 2. The patients (Tier 2) were directly responsible for their mobility footprint, which represents 33.2% of the total. External stakeholders (external laboratories, transport services = Tier 3) were responsible for the remaining 11.0%, which was mainly (89.0%) due to transport activities (couriers).

**Fig. 3:**
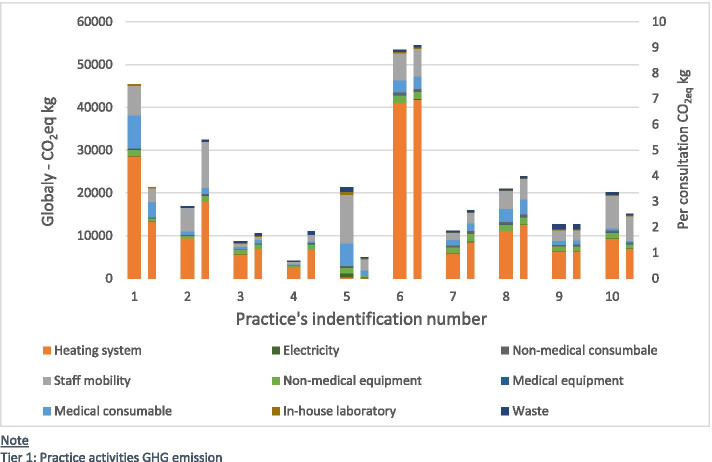
Yearly carbon footprint (tier 1 only) by practice (left/large column) and per consultation (right/small column)

Considering only emissions coming directly from the practice (Tier 1), we described their total and per consultation emissions (Fig. 3). Again, the heating footprint dominated. The practice with highest carbon emissions ranked bottom of the list for both total and per consultation footprints.

For each domain, were extracted those with the lowest and highest footprints per consultation and combined them to establish a theoretical lowest carbon emitting practice and highest carbon emitting practice. This showed a factor of ten difference between the two scenarios.

## Discussion

In this study, the average primary care consultation generated 4.8 CO_2_eq kg. The average practice produced 30.5 CO_2_eq tons overall annually. Transport of staff and patients produced almost half of the total emissions. The heating system was the second emission domain, whereas medical consumables or in-house laboratory had a small impact. Emergency analyses requiring on-call couriers caused 5.7% of the total. Overall, most of the footprint involved domains not directly linked to medical activity. A hypothetical practice conceived by the most efficient characteristics of the ten practices, would produce ten times less CO_2_eq than a hypothetical practice with the least efficient characteristics.

A recent UK study found that the average primary care consultation produces 66 CO_2_eq kg [12], which is ten times that of our results. The main difference between the studies is that the UK study took into account drug prescription (pharmaceuticals supply chain and metered dose inhalers), which represented 60% (3517 out of 5770 CO_2_eq kilotons) of the measured total footprint. Moreover, Swiss and British GP practice organization differ highly, for example in their average consultation times (UK: 9 min., CH: 16 min) [27] and the daily number of consultations, therefore daily patient transport. In addition, in this UK study, patients commuted an average of 13.3 km (8.3 miles) compared to 5.5 km (3.4 miles) in our study, which increases the UK practice footprint. Of note, the localisation of the practice greatly influences its carbon footprint in our study, in that an increase in transport distance from 5.5 to 15 km (distance representative of rural areas) raises the footprint of an average consultation by 56%. Outside of these three aspects, (prescription, consultation times and distance to practices), our results are comparable. This illustrates the importance of reporting variables for each context so that comparisons address the specificities of each setting, for example, mobility habits, average consultation times. It is becoming necessary to standardise measures for primary care variables so that the increasing numbers of studies can be effectively compared.

We chose not to take drug prescription into account, although studies have suggested that this domain accounts for 10 to 22% of the health system footprint [13, 28, 29], and even more in primary care practice as published recently [12]. Primarily, in the Swiss context physicians do not handle prescription alone but alongside other specialists. It would not have been possible, within our design and without access to individual prescription data, to specifically quantify the part of the prescription attributable to primary care. However, physicians can have a direct influence on aspects like choosing lower impact emission supply (for example dry powder inhaler vs metered dose inhalers) or prescribing from manufacturers that seek to improve emissions [30]. Thus, prescription strategy should be set at a higher level. For example, The Nordic organization Sykehusinnkjøp, the NHS and the European Union have developed joint pharmaceutical procurement strategies that include sustainability criteria [16, 31, 32]. These plans aim to push industry to mitigate their carbon footprints. Finally, on a methodological level, as prescriptions were not recorded electronically in some participating practices, estimation of the prescription patterns would have been imprecise. Further, using these individual data would have required ethical clearance, which we did not have. For similar reasons, we did not analyse referrals to secondary care and other emissions than CO_2_eq, such as micro pollutants.

Overall, we found that domains not directly linked to medical activities had the biggest footprints. We could therefore achieve acceptable mitigation without affecting the quality of patient care.

As we found that more than half of the practice emissions come from staff, patient or courier transport, it means that mitigation of a practice’s carbon footprint is grounded primarily in transport organization. While this domain is not directly under the practice’s control, it could ultimately have an impact on the patient’s health. Specifically, directly, in that a long journey means poorer access to primary care, or indirectly, as pollution causes many diseases. Primary care practices could act as a role model for the patient and the community as regards carbon footprints, as they did for tobacco consumption when they demanded smoke-free facilities. Indeed, a dense and local network of primary care practices could decrease the length of the journey a patient needs to make to see his or her doctor and encourage her or him to come by foot. In addition, an effective network of public transportation could prompt staff to commute to work rather than use their car. Finally, the ability to perform urgent laboratory tests within the practice would significantly reduce their carbon footprint, by less usage of the on-call courier.

Among domains not directly linked to medical activities, improving heating systems and optimizing a practice’s occupancy could be high priorities. We noticed that some practices improve significantly their carbon footprints by providing many consultations at the same time. Indeed, a room seldom used but heated weighs significantly on a practice’s footprint. However, even if a well-insulated practice uses less energy and eventually has less expenses, initial investment is very high and can be out of reach of young doctors opening new practices.

In addition, telehealth could be investigated as a carbon mitigation option, to cut down on transportation (patient and staff), which are the top emitters in this study. Indeed, online consultation does not require any travelling. In the other hand, online consultations could raise emissions through increased electronic usage and data storage equipment. Further, patient could still need to travel to do some test or examination. Furthermore, although the current COVID-19 pandemic has shown that primary care can be delivered online in special and emergency times [33], it is unclear if and how habits will change in the near future. In addition, telehealth can only partly replace the multifaceted aspects of a clinical encounter. For example, continuity of care has several dimensions, continuity of information, continuity of management and interpersonal continuity. The latter, probably the most important in primary care, may never be achieved through telehealth. In this respect, in 2014, only 12% of the Swiss population chose an insurance model that implied first consultations delivered online. Moreover, 80.1% lived less than 20 min away from their primary care practice (independent of mean of travel and area) [16].

Medical consumables, often suspected as having a large impact, had, in fact, a small footprint, mainly from bandages and gauze pads. In this respect, questions about the packaging, compounds, and necessity for systematic sterilisation could be raised.

The study has limitations including the fact that only 10 practices were included; however, they were relatively representative of the variety of practices that can be found in Switzerland [22]. Furthermore, the high difference between the best- and worst-case practice emissions indicates that we probably captured a large spectrum of practices.

It was outside the scope of this study to estimate the respective contributions of primary care and other parts of the health system to the overall carbon footprint. Additionally, we could have speculated on the Beneficial effect on emissions of reinforcing prevention, for example favouring active mobility or healthier nutritional habits, important aspects of primary care, but not possible to examine in our calculation.

Finally, this study focused on direct and indirect carbon emission activities in ten primary care practices in Switzerland, allowing a first glance into their carbon footprints in a truly bottom-up approach. The method could be extended to other settings to produce comparable results. Also, due to its importance, drug prescription should be evaluated in future work. Nevertheless, our results already provide important leads on sources of carbon emission in practices, which can be translated into recommendations for primary care physicians and policy makers.

## Conclusion

Primary care practices could significantly lower their carbon footprint, without decreasing the quality of care or changing medical habits. Indeed, main mitigation options would involve heating and transport. The primary care sector, while handling the vast majority of common health problems, is responsible for only a tiny part of the entire health care sector footprint. Even so, implementation of good environmental habits could decrease a practice’s footprint by up to a factor ten, and could serve as role model to other domains of health care services.

## Data Availability

Data are available upon reasonable request:dmf.info@unisante.ch. As some data involve practice business, a data sharing agreement from their owner may be necessary in some cases.

## Abbreviations

GHG: Greenhouse Gas
LCA: Life cycle analysis
CO2eq: Carbon dioxide equivalent
IPCC: Intergovernmental Panel on Climate Change

## References

[1] Intergovernmental Panel On Climate Change (IPCC): Climate change 2014: synthesis report. Contribution of working groups I, II and III to the fifth assessment report of the intergovernmental panel on climate Change 2014.

[2] Wise J (2021). Climate crisis: over 200 health journals urge world leaders to tackle “catastrophic harm”. BMJ.

[3] Health and Climate | Richard Horton [https://www.youtube.com/watch?v=YEVGNeneYug].

[4] Watts N, Amann M, Arnell N, Ayeb-Karlsson S, Beagley J, Belesova K (2021). The 2020 report of the lancet countdown on health and climate change: responding to converging crises. Lancet.

[5] Watts N, Amann M, Arnell N, Ayeb-Karlsson S, Belesova K, Berry H (2018). The 2018 report of the lancet countdown on health and climate change: shaping the health of nations for centuries to come. Lancet (London, England).

[6] Watts N, Amann M, Arnell N, Ayeb-Karlsson S, Belesova K, Boykoff M (2019). The 2019 report of the lancet countdown on health and climate change: ensuring that the health of a child born today is not defined by a changing climate. Lancet.

[7] Malik A, Lenzen M, McAlister S, McGain F (2018). The carbon footprint of Australian health care. Lancet Planetary Health.

[8] Wu R (2019). The carbon footprint of the Chinese health-care system: an environmentally extended input-output and structural path analysis study. Lancet Planetary Health.

[9] Pichler P-P, Jaccard IS, Weisz U, Weisz H. International comparison of health care carbon footprints. Environ Res Lett. 2019;14:064004.

[10] Karliner J, Slotterback S, Boyd R, Ashby B, Steele K: Health care's climate footprint. In. Edited by Series C-shc: Health care without harm & ARUP; 2019.

[11] Nansai K, Fry J, Malik A, Takayanagi W, Kondo N (2019). Carbon footprint of Japanese health care services from 2011 to 2015. Resour Conserv Recycl.

[12] Tennison I, Roschnik S, Ashby B, Boyd R, Hamilton I, Oreszczyn T (2021). Health care's response to climate change: a carbon footprint assessment of the NHS in England. Lancet Planetary Health.

[13] Eckelman MJ, Sherman J (2016). Environmental impacts of the U.S. health care system and effects on public health. PLoS One.

[14] UNEP/GRID-Geneva & University of Geneva (2015). Environmental limits and Swiss footprints based on planetary boundaries.

[15] Go Green for 2015 - Top Tips for General Practice Teams [https://sustainablehealthcare.org.uk/news/2014/12/go-green-2015-top-tips-general-practice-teams].

[16] Delivering a ‘Net Zero’ National Health Service [https://www.england.nhs.uk/greenernhs/].

[17] Tomson C (2015). Reducing the carbon footprint of hospital-based care. Future Hosp J.

[18] Purohit A, Smith J, Hibble A (2021). Does telemedicine reduce the carbon footprint of healthcare?. A systematic review. Future Healthc J.

[19] Green Impact for Health toolkit [https://www.greenimpact.org.uk/giforhealth].

[20] My Green Doctor [www.mygreendoctor.org/].

[21] Andrews E, Pearson D, Kelly C, Stroud L, Rivas Perez M (2013). Carbon footprint of patient journeys through primary care: a mixed methods approach. Br J Gen Pract.

[22] Senn N, S Ebert and C. Cohidon. La médecine de famille en Suisse. Analyse et perspectives sur la base des indicateurs du programme SPAM (Swiss Primary Care Active Monitoring) (Obsan Dossier 55). 2016.

[23] ISO 14040:2006(en) - Environmental management — Life cycle assessment — Principles and framework. 2016.

[24] Fazio S, Castellani V, Sala S, Schau E, Secchi M, Zampori L, Diaconu E. Supporting information to thecharacterisation factors of recommended EF Life Cycle Impact Assessment methods: New methods and differences with ILCD, EUR 28888 EN. Luxembourg: Publications Office of the European Union; 2018. 10.2760/671368, JRC109369.

[25] Hostettler K. Peu de femmes aux postes de cadre. Statistique médicale 2018 de la Fédération des médecins suisses. Bull des médecins suisses. 2019;100(12):411–16.

[26] ECOINVENT - Life cycle inventory database [https://www.ecoinvent.org/].

[27] Irving G, Neves AL, Dambha-Miller H, Oishi A, Tagashira H, Verho A (2017). International variations in primary care physician consultation time: a systematic review of 67 countries. BMJ Open.

[28] Eckelman MJ, Sherman JD, MacNeill AJ (2018). Life cycle environmental emissions and health damages from the Canadian healthcare system: an economic-environmental-epidemiological analysis. PLoS Med.

[29] Carbon Hotspots update for the health and care sector in England 2015 (www.sduhealth.org.uk/report).

[30] Sherman JD, McGain F, Lem M, Mortimer F, Jonas WB, MacNeill AJ (2021). Net zero healthcare: a call for clinician action. BMJ.

[31] Pharmaceutical Strategy of The Norwegian Hospital Procurement Trust (Sykehusinnkjøp HF) [https://sykehusinnkjop.no/pharmaceutical-strategy-of-the-norwegian-hospital-procurement-trust-sykehusinnkjop-hf-#a-short-description-of-the-focus-areas].

[32] Hernández Olivan P, Jósa V, Long A: Reducing the carbon footprint of healthcare through sustainable procurement. Health Care Without Harm (HCWH) Europe 2018.

[33] Savoy M, Haller DM, Rytz R, Mueller Y (2021). COVID-19 et téléconsultations. Gestion de la crise dans les cabinets de médecins du réseau Sentinella selon Une étude observationnelle. Prim Hosp Care Med Int Gen.

